# Metal Artifact Reduction in Oral Cavity MDCT Using Dental Overlays and the O-MAR Algorithm

**DOI:** 10.3390/diagnostics16172881

**Published:** 2026-09-07

**Authors:** Svetlana Antic, Maja Lezaja Zebic, Jovana Kuzmanovic Pficer, Djurdja Bracanovic, Aleksa Janovic, Biljana Markovic Vasiljkovic, Drago B. Jelovac

**Affiliations:** 1 Center for Radiological Diagnostics, School of Dental Medicine, University of Belgrade, Rankeova 6, 11000 Belgrade, Serbiaaleksa.janovic@stomf.bg.ac.rs (A.J.); biljana.markovic.vasiljkovic@stomf.bg.ac.rs (B.M.V.); 2 Implant Research Center, School of Dental Medicine, University of Belgrade, 11000 Belgrade, Serbia; maja.zebic@stomf.bg.ac.rs; 3 Department for Medical Statistics and Informatics, School of Dental Medicine, University of Belgrade, 11000 Belgrade, Serbia; jovana.kuzmanovic@stomf.bg.ac.rs; 4 Clinic for Maxillofacial Surgery, School of Dental Medicine, University of Belgrade, dr Subotica 8, 11000 Belgrade, Serbia

**Keywords:** metal artifacts, MDCT, dental materials, dental overlays

## Abstract

**Background/Objectives**: To evaluate the impact of dental laboratory material overlays on metal artifact reduction in multidetector computed tomography (MDCT) of the oral cavity across different CT protocols and reconstruction algorithms. **Methods**: An anthropomorphic mandible phantom was constructed using extracted human teeth, metal-ceramic restorations, and ballistic gelatin as a soft-tissue analog. Six dental materials (Elite HD+, Vonflex S Putty, Tropicalgin, MB Wax 1, Zetaplus, and COE-PAK) were tested as overlays. MDCT imaging was performed following low-dose and standard protocols with primary and O-MAR reconstructions, yielding 28 datasets. Two radiologists independently assessed artifact indices (AIs), overall image quality (OIQ), and spherical marker visibility (SMV), with excellent inter-observer agreement (ICC = 0.985). **Results**: Vonflex S Putty model consistently demonstrated the lowest AIs and highest OIQ scores, while maintaining stable performance across both protocols and reconstructions. Elite HD+ and Zetaplus also showed favorable results, while COE-PAK and MB Wax 1 frequently underperformed. Tropicalgin exhibited high AIs with primary reconstruction but improved markedly with O-MAR. SMV analysis confirmed Vonflex S Putty as the only material to consistently outperform the control under low-dose conditions. **Conclusions**: Silicone-based overlays, particularly Vonflex S Putty, are effective in reducing metal artifacts and improving image quality in MDCT. Overlay selection, combined with MAR algorithms such as O-MAR, represents a complementary strategy for optimizing diagnostic accuracy in patients with metallic restorations.

## 1. Introduction

Multidetector Computed Tomography (MDCT) is widely used in head, neck, and dento-maxillofacial (DMF) imaging due to its availability, high spatial resolution, and rapid performance. Compared to Cone Beam CT (CBCT), which is commonly used in DMF diagnostics, MDCT offers superior soft-tissue visualization and enables contrast-enhanced studies. This makes it particularly valuable for assessing oral cavity cancers (OCCs), especially when bone infiltration or regional lymph node involvement must be evaluated—both critical for loco-regional control and disease-free survival [1].

A major limitation of DMF imaging, however, is the presence of artifacts from dental metal restorations, which affects CBCT, MDCT, and Magnetic Resonance Imaging (MRI) alike. Artifacts—defined as image features or discrepancies not present in the actual object—can obscure anatomical and pathological structures within the region of interest (ROI) [2,3,4]. Metal artifacts arise from physical phenomena such as beam hardening, photon starvation, scatter, and edge effects—all related to X-ray attenuation and scattering properties of high-density materials [2,3,5]. Each of the listed physical phenomena overcomes the capabilities of the reconstruction algorithm to cope with it, resulting in dark bands, white linear streaks, and graininess of the reconstructed image [2,3].

Various strategies have been developed to reduce metal artifacts in MDCT. Conventional acquisition adjustments (e.g., higher tube voltage/current, extended CT scale, thin-section collimation, specialized kernels) often yield limited success and may increase the radiation dose [5,6]. Modern CT systems incorporate filtration, calibration, and correction software, while post-processing metal artifact reduction (MAR) techniques such as projection interpolation, iterative reconstruction, and deep learning enhanced algorithms have shown promise [6,7,8,9,10]. Dual-energy CT (DECT) mitigates beam hardening through virtual monochromatic imaging and material separation at different energies, without significantly raising the radiation dose [6]. Photon-counting CT (PCCT) further improves spatial resolution and artifact reduction via high-energy bin reconstruction and virtual mono-energetic imaging. Although combining DECT or PCCT with MAR techniques is encouraging, these approaches require specialized hardware not yet widely available [5,11,12,13]. Despite these advances, metal artifacts remain a persistent challenge, particularly when it comes to evaluating oral cavity lesions in patients with metal dental restorations. Each contribution to this field is therefore of great importance.

However, specific anatomic conditions in the oral cavity enabled testing a unique approach to metal artifact reduction, based on the potential of dental overlays to absorb scattered radiation and mitigate beam hardening. A few recent studies have examined the effects of dental overlays made of different prosthodontic impression materials on metal artifacts in dental CBCT [2,14]. To the best of our knowledge, there are no studies addressing the potential effects of such dental overlays on metal artifacts in MDCT.

Therefore, the aim of this study was to assess the impact of dental laboratory material overlays on metal artifacts in MDCT examinations across different CT protocols and reconstruction algorithms, including MAR.

## 2. Materials and Methods

### 2.1. Ethical Approval

This study was conducted in accordance with the ethical principles of the World Medical Association Declaration of Helsinki (1975), as amended in 2000, and received approval from the Institutional Ethical Committee (Decision No. 36/21, 15 May 2024).

### 2.2. Phantom Design

**Figure 1:**
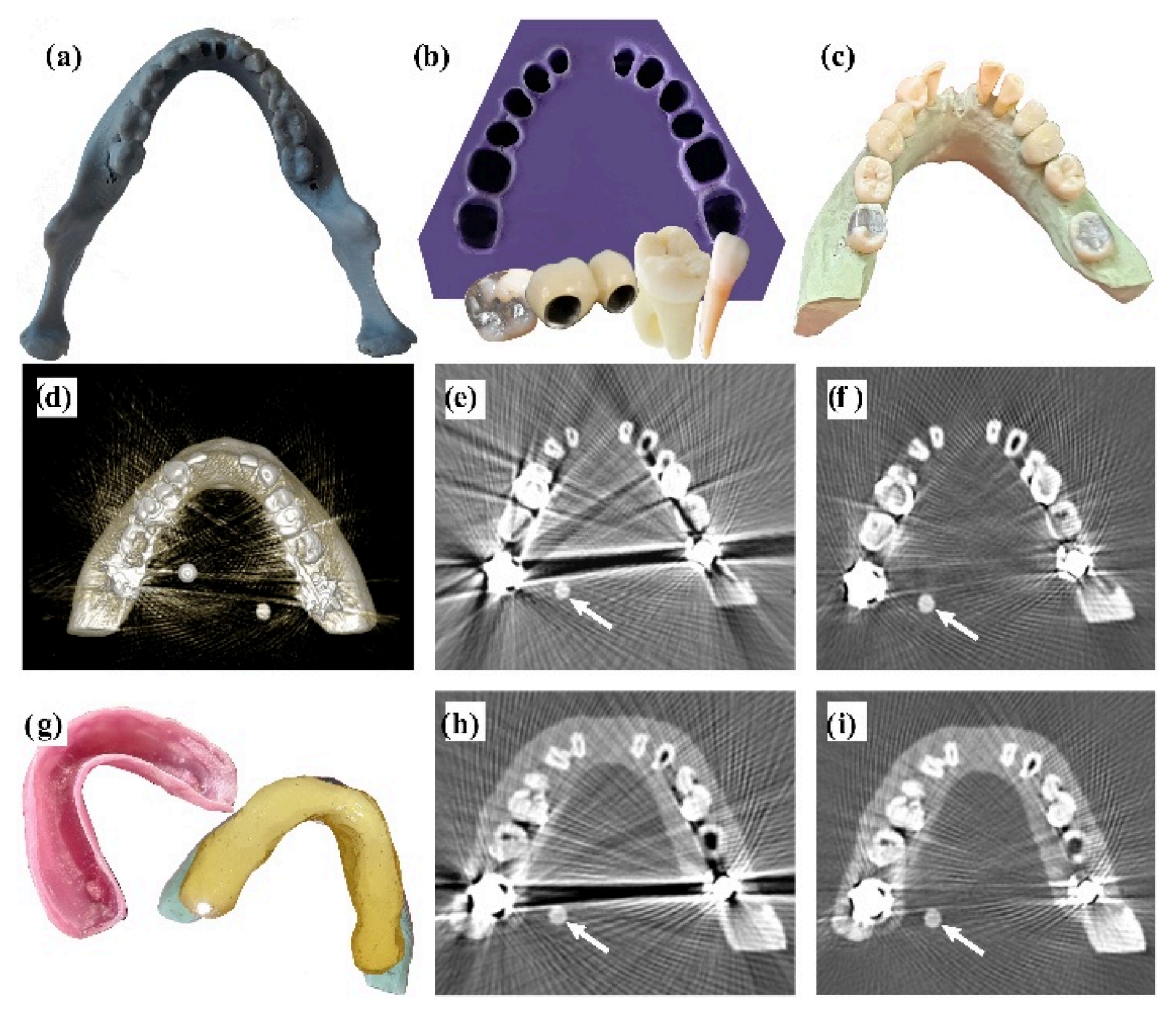
Anthropomorphic phantom construction and analysis phases: (**a**) 3D-printed plastic replica of a dentate mandible; (**b**) silicon duplicate mold; (**c**) plaster model of the jaw with inserted human teeth; (**d**) 3D reconstruction of the scanned control model immersed in ballistic gelatin; (**e**) axial section of the control model with primary reconstruction; (**f**) axial section of the control model with O- MAR reconstruction; (**g**) impression tray for overlay material; (**h**) axial section of a tested model with primary reconstruction; (**i**) axial section of a tested model with O- MAR reconstruction; an arrow points to a spherical marker in the images (**e**,**f**,**h**,**i**).

Intact human teeth—specifically four incisors and four molars—of patients referred for extraction due to dental or medical indications were collected at the Dental University Clinic and employed in the phantom model construction of a mandible for the purpose of this study. Two molars were restored with extensive amalgam fillings (EXTRACAP^®^ NON GAMMA2, Profarm, Belgrade, Serbia) extending toward the root canal openings and across the proximal surfaces. The remaining two molars and four anterior teeth were used in their original, unaltered state. Additionally, two metal-ceramic prosthodontic restorations were incorporated, one on each side of the jaw in the premolar region. The external contour of the mandible and the anatomical positioning of the teeth were modeled using a 3D-printed plastic replica of a dentate mandible (Figure 1a). A duplicate mold was created using silicone (Elite Double, Zhermack, Badia Polesine, Italy) (Figure 1b). The extracted human teeth—with or without restorations—and the metal-ceramic prosthodontic units were positioned within the silicone mold at designated tooth sites. Concurrently, the remaining bone area of the model was cast using type IV super-hard dental plaster (megaUNISTONE, Megadental GmbH, Büdingen, Germany) (Figure 1c).

An impression tray (POLIDENT Unitray LC, Volčja Draga, Slovenia) was fabricated with a uniform 3 mm clearance from the tooth surfaces to ensure consistent thickness across all tested materials (Figure 1g). This replication guide was subsequently employed to mold six distinct dental laboratory materials; each applied to uniformly envelop the coronal portions of the teeth at the specified thickness:

1. Addition Silicone (Elite HD+);
2. Addition Silicone (Vonflex S Putty);
3. Alginate (Tropicalgin);
4. Pink wax (MBwax1);
5. Condensation Silicon (Zetaplus);
6. Zinc-oxide paste (COE-PAK).

Ballistic gelatin was selected as a soft tissue analog due to its recent recognition in dental maxillofacial radiology and its demonstrated subjective and objective resemblance to native soft tissues [15]. The gelatin was prepared by combining 48 g of colorless, unflavored gelatin with 200 mL of glycerin and 500 mL of water [15,16]. These proportions had previously been validated in our recent CBCT study [14], thus ensuring that the final product exhibited appropriate consistency, stability at room temperature, homogeneity, and a density lower than that of dental and osseous structures. This was crucial for facilitating clear visualization of imaging artifacts. The mixture was boiled for 25 min and subsequently cooled to room temperature for up to 20 min. The mandible phantom was placed within a plastic mold into which the ballistic gelatin was poured to simulate surrounding soft tissues. Two plaster spherical bodies, each 4 mm in diameter, were embedded in the gelatin to serve as positional controls and section markers (Figure 1d). The spherical body located 10 mm lingual to the right molar region and containing two sub-millimeter grains of varying size and density was employed for a qualitative analysis (Figure 1e,f,h,i).

### 2.3. MDCT Image Acquisition

The finalized phantom was subjected to CT imaging using a Philips Ingenuity 64 CT unit (Philips Healthcare, Cleveland, OH, USA), with thin-section collimation (0.67 mm) and 136 mm field of view, under two distinct scanning protocols: a low-dose protocol (120 kV, 280 mA) and a standard protocol (120 kV, 468 mA). The phantom’s position was consistently marked to ensure reproducibility across all scans. The aforementioned scanning protocols were subsequently repeated six additional times. Each time, a different dental impression material was applied to cover the teeth.

### 2.4. Image Processing

Each dataset was evaluated both in its original form and after applying the O-MAR (Orthopedic Metal Artifact Reduction) reconstruction algorithm (Figure 1e,f,h,i). The reconstruction slice thickness was 0.67 mm. A total of 28 acquired datasets were ultimately included in the analysis.

### 2.5. Quantitative Analysis

**Figure 2:**
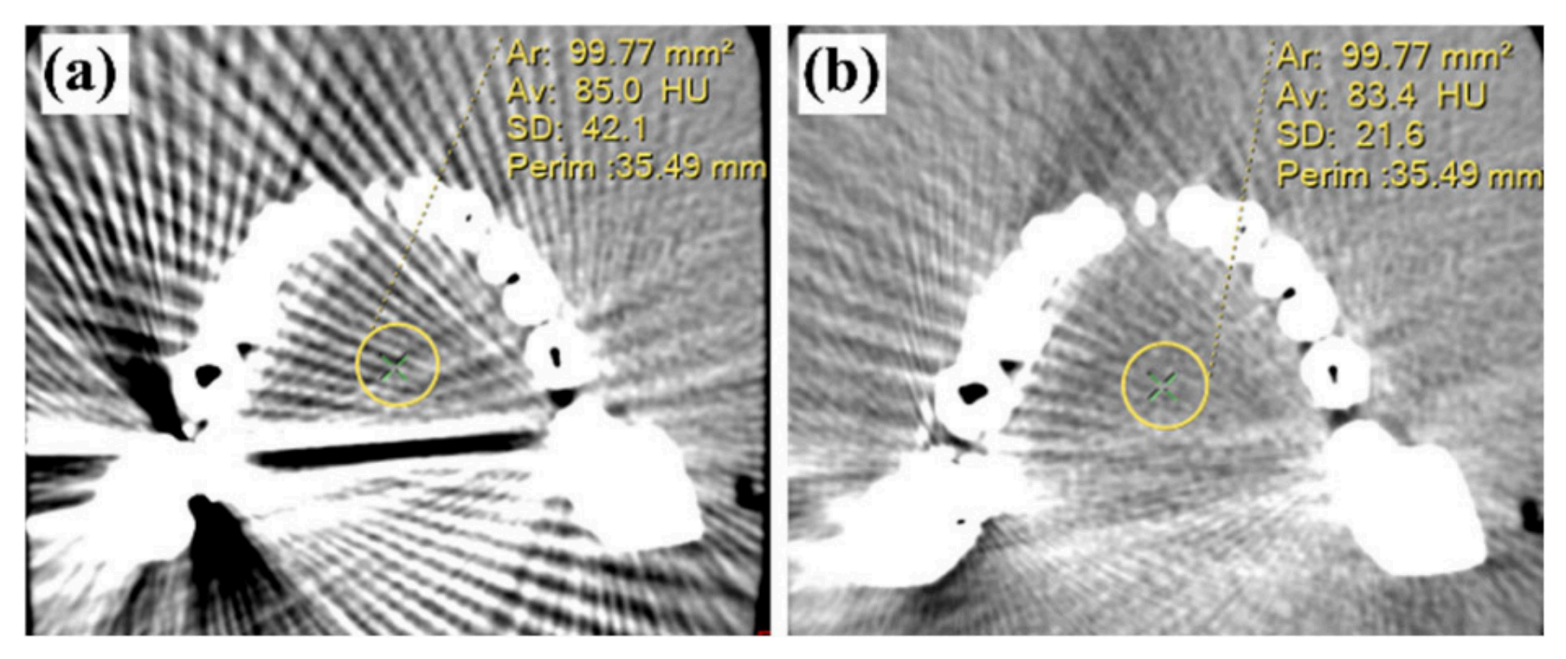
Circular region of interest (ROI) placed within the simulated soft-tissue region of the mandibular arch, with measurable mean CT values and standard deviations (SD) (**a**) axial section of the control model with primary reconstruction; (**b**) axial section of the control model with O- MAR reconstruction.

For the quantitative analysis, a soft-tissue reconstruction kernel (Standard, B) was applied as literature indicates that such kernels provide a more realistic representation of image noise and yield more stable CT values with reduced variability [17]. After one week of training, two experienced radiologists independently performed a quantitative image analysis at a Philips workstation. A circular region of interest (ROI), with an approximate surface area of 1 mm^2^, was manually placed within the simulated soft-tissue region of the mandibular arch, midway between the second premolars (Figure 2). The mean CT values, expressed in Hounsfield units (HU) and their standard deviation (SD), were obtained by replicating the same ROI across four consecutive axial slices in all datasets, including both primary reconstructions and those processed with O-MAR. This was conducted at identical slice levels using a copy-and-paste function. The ROIs were consistently placed in the same anatomical areas across all datasets. The evaluation was performed using the Artifact Index (AI), defined as:

$$\mathrm{AI}\;=\;\sqrt{SDa^{2}-SD{ref}^{2}}$$

where SD_a_ represents the standard deviation of CT values within a ROI affected by artifacts, while SD_ref_ denotes the standard deviation of the CT values in a relatively homogenous reference ROI outside the artifact region. This definition isolates the variance attributable to the artifact component by subtracting the noise variance from the total variance [17].

### 2.6. Qualitative Analysis

For the qualitative assessment of metal artifacts, a bone reconstruction kernel (YA) was applied as sharper kernels enhance edge definition and allow streak and beam-hardening artifacts to be more clearly visualized. This approach was chosen exclusively for the technical evaluation of artifacts in the phantom study, motivated by the phantom design, which incorporated a spherical body composed of compact gypsum-based material with granular inclusions rather than simulated soft tissue lesions. The sharper reconstruction facilitated enhanced edge definition and enabled precise localization of pronounced artifacts.

After dedicated training, two experienced radiologists independently conducted the qualitative analysis of all datasets. The evaluations were performed using computers equipped with 24-inch LCD monitors (2560 × 1440 resolution) in a silent and low-light environment. Overall image quality was assessed through all slices using a five-point Likert scale [18] (1 = non-diagnostic; 2 = poor, diagnosis doubtful; 3 = acceptable, enabling diagnosis; 4 = good, confidently diagnostic; 5 = excellent). In addition, the radiologists evaluated the visibility of a circular plaster body containing two sub-millimeter grains of differing size and density, embedded in ballistic gelatin, and positioned 10 mm lingual to the molar region, using selected representative slices (spherical marker on Figure 1e,f,h,i). This was likewise rated on a five-point Likert scale (1 = not identifiable; 2 = identifiable without detail; 3 = assessable in most details; 4 = assessable in detail; 5 = perfectly delineated). After a one-week interval, each radiologist repeated the measurements.

### 2.7. Statistical Analysis

The data were analyzed using SPSS statistical software (version 22; SPSS Inc., Chicago, IL, USA). Numerical data were expressed as means with standard deviations (SD), as well as medians with minimum and maximum values. The normality of distribution of continuous variables was tested using the Kolmogorov–Smirnov test. For normally distributed data, one-way ANOVA with Bonferroni post hoc analysis was applied to determine statistically significant differences between materials, whereas the Kruskal–Wallis test with Mann–Whitney U test was used for non-parametric data. The level of statistical significance was set at *p* ≤ 0.05. Inter-rater reliability between the two radiologists was assessed using the Intraclass Correlation Coefficient (ICC) based on a two-way random-effects model with absolute agreement. Single-measure ICC values with 95% confidence intervals (CI) were reported.

## 3. Results

### 3.1. Quantitative Analysis

Inter-observer agreement between the two radiologists was excellent (ICC = 0.985, 95% CI 0.980–0.989, *p* < 0.001). Artifact indices (AIs) of the tested models differed significantly across both CT protocols and reconstruction algorithms (Table 1, a: comparisons among all the models). A post hoc analysis was conducted to compare each tested model containing a tooth overlay with the control (Table 1, b: post hoc analysis).

Under the low-dose protocol with primary reconstruction (Table 1), all tested models exhibited significantly lower AIs compared to the control, with the lowest values recorded for Vonflex S Putty. With regard to O-MAR reconstruction, AIs were markedly reduced compared to primary reconstructions. Although most models (except COE-PAK) significantly differed from the control, only Vonflex S putty and Tropicalgin demonstrated lower values than the control.

Similarly, under the standard protocol with primary reconstruction, nearly all models (except Tropicalgin) showed significantly lower AIs when compared to the control, and the lowest value was once again recorded for the Vonflex S Putty model. With the O-MAR reconstruction algorithm, AIs were substantially reduced across all models. Vonflex S Putty and MB wax1 showed no significant differences relative to the control. Tropicalgin exhibited significantly lower AIs, while the remaining models displayed higher AI values compared to the control.

An interesting finding was that certain materials such as Tropicalgin, which exhibited the highest AIs with primary reconstruction, showed the lowest AI values after O-MAR application. Notably, Vonflex S Putty was the only material that consistently maintained a stable trend of relatively low AIs across CT protocols and reconstruction algorithms (Table 1).

### 3.2. Qualitative Analysis

The inter-class correlation coefficient (ICC) for overall image quality scores assigned by both radiologists was high (ICC = 0.874; 95% CI, 0.833–0.904; *p* < 0.001).

The ICC for spherical marker visibility scoring was also high (ICC = 0.889; 95% CI, 0.827–0.905; *p* < 0.001).

Regarding the overall image quality (Table 2), under the low-dose protocol and with both reconstruction modalities (primary and O-MAR), three of the tested models: Elite HD+, Zetaplus and Vonflex S Putty achieved significantly higher scores compared to the control, with Vonflex S Putty recording the highest mean score. In contrast, with O-MAR reconstruction, MB Wax 1 and COE-PAK exhibited significantly lower scores relative to the control.

Under the standard protocol with primary reconstruction, all tested models significantly differed from the control. Vonflex S Putty again demonstrated the highest scores, whereas MB Wax 1 showed lower scores than the control. With O-MAR reconstruction, most of the tested models (except MB Wax 1) significantly differed from the control: Vonflex S Putty, Elite HD+ and Zetaplus demonstrated higher scores, while COE-PAK exhibited a lower mean score compared to the control (Table 2).

Concerning spherical marker visibility (Table 3), under the low-dose protocol with primary reconstruction, most of the tested models (except Zetaplus and Elite HD+) significantly differed from the control, but only Vonflex S Putty achieved higher scores. A similar trend was observed with O-MAR reconstruction under the low-dose protocol, except that Elite HD+ exhibited significantly lower scores than the control.

Under the standard protocol with primary reconstruction, only Vonflex S Putty and COE-PAK did not differ significantly from the control, while all other models displayed significantly lower scores. With O-MAR reconstruction under standard protocol, only COE-PAK significantly differed from the control, and in this case, it demonstrated lower scores (Table 3).

**Figure 3:**
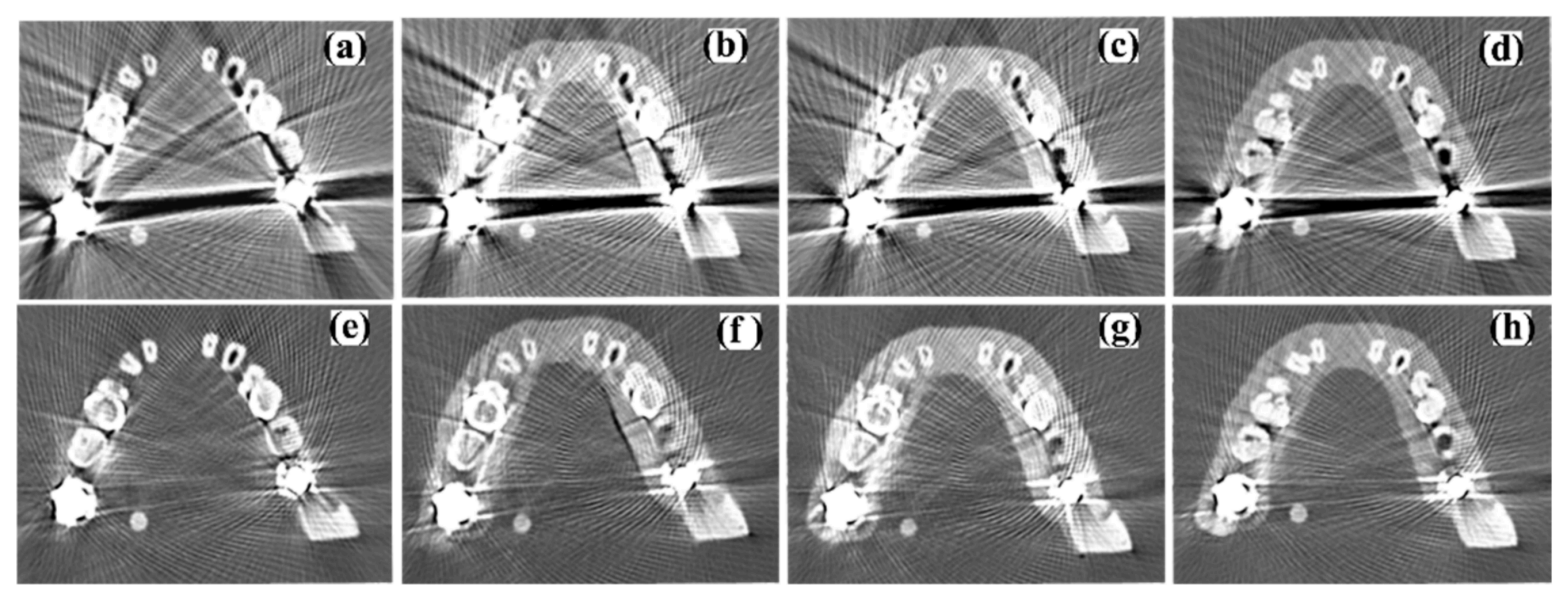
Differences between the control (**a**,**e**) at two different reconstruction algorithms (primary—upper row, O-MAR—lower row) and the tested materials: Elite HD+ (**b**,**f**), Zetaplus (**c**,**g**) and Vonflex S Putty (**d**,**h**).

Figure 3 presents three of the tested models (Elite HD+, Zetaplus and Vonflex S Putty), with the best results in the qualitative analysis compared to the control.

## 4. Discussion

Although modern dentistry increasingly favors esthetic, non-metallic restorative materials, even light metals used in certain restorations, as well as titanium implants, continue to exert a detrimental impact on MDCT images. Consequently, metal artifacts in oral cavity MDCT scans remain a significant challenge.

Various strategies for metal artifact reduction in MDCT have been investigated, with the primary focus on CT acquisition parameters, protocol optimization, and reconstruction algorithms [5,6,7,8,9,10,11,12,13]. Most metal artifact reduction (MAR) algorithms were originally developed to address artifacts from orthopedic implants [6], including O-MAR applied in the present study. In the case of dental restorations, however, the specific anatomy of the oral region allows for testing a unique approach—the use of dental overlays designed to absorb scattered radiation, reduce beam-hardening effects, and thereby minimize artifacts. Inspired by protective shielding techniques in therapeutic radiology [19,20], we had previously analyzed the same set of materials used for dental overlays in a CBCT study, which provided valuable insights into their radiographic behavior [14]. With the exception of zinc-oxide (COE-PAK), which is primarily used to aid healing, the materials tested in the current study are commonly employed as radiographic guides and/or interocclusal record materials in CBCT-guided implant procedures, and their radiodensity characteristics have already been reported [21,22,23]. In MDCT, overlays exhibiting higher radiodensity than the adjacent soft tissues but lower than metallic restorations may reduce beam hardening and absorb scattered photons, thereby diminishing artifacts. To be effective, however, the material must remain relatively homogeneous to avoid introducing new distortions.

The present study analyzed the impact of dental overlays, made of six different dental lab materials, on the extent of metal artifacts both quantitatively and qualitatively across two different CT protocols and reconstruction algorithms. Inter-observer agreement was excellent, confirming the reliability of the methodology and strengthening confidence in the reported findings. Jaw anatomy was replicated using soft-tissue simulation to construct an anthropomorphic phantom that closely reflects clinical conditions. Ballistic gelatin was used as a soft tissue simulator since its density approximates muscle tissue (1060 kg/m^3^) but is lower than the density of dental hard tissues and bone, which enabled clear visualization of artifacts. Its physicochemical properties account for its widespread use as a surrogate for human tissue, particularly in forensic applications [15,16]. Metal restorations were placed bilaterally in the mandibular arch to reproduce realistic scenarios and increase artifact complexity through overlapping effects.

Unlike most clinical studies that employ soft tissue windows for both quantitative and qualitative assessment, our phantom setup required a different reconstruction strategy. The phantom incorporated a spherical marker composed of compact gypsum-based material with granular inclusions rather than simulated soft tissue lesions, which justified the use of a bone kernel for the purpose of qualitative analysis to accentuate fine structural details and delineate zones of pronounced artifacts. This methodological choice ensured that artifacts were captured in their most visible form, thus facilitating qualitative assessment and precise localization. The dual approach—bone window for qualitative visualization and soft-tissue window for quantitative analysis—enabled artifacts to be documented in their most pronounced form while maintaining methodological rigor and ensuring comparability with prior studies.

Across both CT protocols, statistically significant differences in artifact indices (AIs) were observed among the tested models, underscoring the influence of overlay material composition on artifact severity. The low-dose protocol yielded results broadly comparable to the standard protocol, suggesting that dose reduction may not compromise the evaluation of artifacts. However, formal equivalence analysis and assessment of diagnostic accuracy are required to support these findings, and should be assessed in future research.

The reconstruction algorithm exerted a profound effect on artifact expression. As expected, O-MAR reconstruction drastically reduced AIs compared to primary reconstructions, confirming its effectiveness in mitigating metal artifacts. Nevertheless, material-specific differences persisted even after O-MAR application, indicating that reconstruction alone cannot fully neutralize the impact of certain materials. An intriguing observation was that Tropicalgin, which exhibited the highest AIs among the tested models with primary reconstruction, showed the lowest values after O-MAR. This suggests that O-MAR is most effective when baseline artifacts are pronounced, providing a disproportionately greater benefit in such cases.

Vonflex S Putty consistently maintained a stable trend of relatively low AIs across protocols and reconstruction algorithms. The only exception was with O-MAR reconstruction under the standard protocol as its AI values did not significantly differ from the control, while nearly all other models (except the previously mentioned Tropicalgin) exhibited higher AIs. This stability highlights Vonflex S Putty as the most artifact-resistant material with predictable performance regardless of the reconstruction strategy.

In qualitative assessments, Vonflex S Putty also achieved the highest overall image quality (OIQ) scores across protocols and reconstruction algorithms, reinforcing its favorable imaging characteristics. By contrast, MB Wax 1 and COE-PAK mostly exhibited lower OIQ scores relative to the control, suggesting limited suitability as overlay materials in MDCT. Spherical marker visibility further highlighted material-specific effects: Vonflex S Putty was the only material to consistently display higher scores than the control under the low-dose protocol, while other overlay materials displayed lower scores. Under the standard protocol with primary reconstruction, only Vonflex S Putty and COE-PAK did not differ significantly from the control, while all other models demonstrated reduced marker visibility. This indicates that a higher radiation dose alone does not guarantee improved performance. Therefore, material composition remains a critical determinant. With O-MAR reconstruction under the standard protocol, only COE-PAK significantly differed from the control, and in this case, it exhibited lower scores, further confirming its limited suitability as an overlay. Taken together, these findings emphasize that overlay materials influence not only artifact indices and overall image quality but also the visibility of fine radiographic details such as spherical marker in our case. Vonflex S Putty consistently demonstrated superior performance, thus reinforcing its role as the most reliable material across conditions. Conversely, COE-PAK frequently underperformed, reflecting its inhomogeneity and limited capacity to improve diagnostic visualization.

The present MDCT study confirms and extends the observations from our previous CBCT investigation [14], highlighting the pivotal role of silicone-based materials in artifact reduction. Once again, Vonflex S Putty, Elite HD+, and Zetaplus emerged as the most consistent performers. Vonflex S Putty, in particular, maintained a stable trend of low artifact indices across CT protocols and reconstruction algorithms in MDCT, echoing its previously observed homogeneity and favourable radio-density (higher than soft tissue simulator, but lower than a metallic restoration) in CBCT. Elite HD+ and Zetaplus also demonstrated improved image quality, which is consistent with their classification as addition (A) and condensation (C) silicones, respectively. These findings align with Hinchy et al. [2] who reported that A-silicones such as Blu-mousse exhibited minor variance in grayscale values among the tested materials, albeit without statistical significance. Fonseca et al. [21] reported that A-silicones can differ in their consistency (light of heavy-body materials), but their radio-density primarily depends on added high-atomic-number elements. Platinum, acting as a catalyst, and palladium, preventing hydrogen release, along with silica fillers create a stable and homogenous matrix. In contrast, when it comes to C-silicones such as Zetaplus, both filler type and volume fraction significantly influence radiodensity producing regions of differing absorption, and increasing the likelihood of localized distortions. Additionally, C-silicones release ethyl alcohol as a by-product, which leads to shrinkage and heterogeneity within the material [24]. This phenomenon can explain lower performance in artifact reduction compared to A-silicones. COE-PAK which displayed high inhomogeneity and MB Wax 1 with its lower radio-density and inadequacy in filling undermined areas again proved unsuitable. These shortcomings were reflected in both quantitative indices and qualitative assessments of image quality and marker visibility.

Collectively, the consistency between CBCT and MDCT findings strengthens the conclusion that A-silicone materials, particularly Vonflex S Putty, are the most promising candidates for use as dental overlays in artifact reduction. Their radiodensity profile, homogeneity, and ease of clinical handling make it suitable for minimizing the detrimental impact of metallic restorations on CT imaging. The observation that O-MAR reconstruction disproportionately benefits high-artifact materials (e.g., Tropicalgin) further suggests that the overlay selection and the reconstruction strategy should be considered as complementary approaches in optimizing diagnostic image quality which is critical for diagnostic accuracy.

From a clinical perspective, the application and choice of overlay material may substantially influence MDCT image quality in patients with metallic restorations. The application procedure requires minimal chairside time and is well tolerated by patients, which supports the potential integration of overlay use into everyday diagnostic practice. Materials associated with lower AIs and higher OIQ scores, such as Vonflex S Putty, could minimize interference with radiological evaluation and improve diagnostic confidence. Conversely, materials prone to higher AIs or reduced marker visibility may obscure critical anatomical details, particularly in complex cases requiring precise visualization. The observation that O-MAR reconstruction disproportionately benefits high-artifact materials suggests that reconstruction strategy should be tailored to the expected severity of artifacts.

This study has several limitations, notably the use of phantom models, which may not fully replicate clinical conditions. Patient-based studies are warranted to validate these findings and to explore whether similar trends are observed in vivo. Moreover, only one acquisition has been performed for each material-protocol configuration, although the measurements were performed across four consecutive axial slices. Additionally, this study did not include a metal-free control model; thus, smoothing or newly introduced artifacts cannot be fully excluded, although the analysis was specifically focused on artifacts induced by metallic restorations. Future research should consider a metal-free reference model, include multiple acquisitions per material–protocol configuration, as well as to investigate additional reconstruction algorithms and hybrid approaches to further optimize artifact reduction.

## 5. Conclusions

This study has demonstrated that certain dental overlay materials can significantly influence artifact severity and diagnostic quality in MDCT imaging of the oral cavity. Among the tested materials, Vonflex S Putty consistently showed superior performance, while Elite HD+ and Zetaplus also proved effective. In contrast, COE-PAK and MB Wax 1 were limited by inhomogeneity and poor adaptation.

Overall, silicone-based overlays—particularly A-silicones—emerge as the most promising candidates for artifact reduction in MDCT. Their favorable radiodensity profile, homogeneity, and clinical handling properties make them suitable for minimizing the impact of metallic restorations. Combining appropriate overlay selection with MAR algorithms such as O-MAR offers a complementary strategy to optimize diagnostic accuracy.

Future patient-based studies are warranted to validate these phantom-based findings and to explore additional reconstruction techniques for further improvement in dento-maxillofacial imaging.

## Figures and Tables

**Table 1:** Comparison of artifact indices of tested models across CT protocols and reconstruction modalities.

	Low-Dose Protocol				Standard Protocol			
Reconstruction	Primary		O-MAR		Primary		O-MAR	
	AI (SD)	*p*	AI (SD)	*p*	AI (SD)	*p*	AI (SD)	*p*
Model		<0.001 * a		<0.001 * a		<0.001 * a		<0.001 * a
Control	195.47 (6.10)		70.72 (6.93)		182.14 (8.54)		58.23 (8.13)	
Elite HD+	161.27 (3.46)	<0.001 * b	79.66 (1.35)	<0.001 * b	151.21 (12.68)	<0.001 * b	80.94 (6.34)	<0.001 * b
Tropicalgin	188.39 (7.22)	0.003 * b	58.08 (1.08)	<0.001 * b	185.37 (2.72)	0.083	49.31 (1.19)	0.006 * b
MB wax 1	149.92 (4.61)	<0.001 * b	96.02 (4.54)	<0.001 * b	149.00 (12.37)	<0.001 * b	63.70 (1.14)	0.055
Zetaplus	138.10 (4.86)	<0.001 * b	83.77 (1.26)	<0.001 * b	134.56 (3.69)	<0.001 * b	63.99 (0.69)	<0.001 * b
Vonflex S Putty	85.42 (7.46)	<0.001 * b	64.09 (3.86)	<0.001 * b	75.57 (3.05)	<0.001 * b	60.05 (2.89)	0.880
COE- PAK	133.58 (3.77)	<0.001 * b	73.50 (0.86)	0.613	156.47 (2.06)	<0.001 * b	103.92 (15.62)	<0.001 * b

AI = artifact index; SD = standard deviation; a = comparisons among all models, b = post hoc analysis comparing each of the models containing an overlay with the control, * statistically significant.

**Table 2:** Comparison of the models in terms of overall image quality across CT protocols and reconstruction modalities.

	Low-Dose Protocol				Standard Protocol			
Reconstruction	Primary		O-MAR		Primary		O-MAR	
	OIQ Score	*p*	OIQ Score	*p*	OIQ Score	*p*	OIQ Score	*p*
Model		<0.001 * a		<0.001 * a		<0.001 * a		<0.001 * a
Control	1 (1–2) 1.38 ± 0.50		2.50 (2–3) 2.50 ± 0.52		1 (1–2) 1.25 ± 0.45		3 (2–3) 2.75 ± 0.45	
Elite HD+	2 (2–3) 2.06 ± 0.25	<0.001 * b	3 (const.)	<0.001 * b	2 (const.)	<0.001 * b	3 (const.)	0.035 * b
Tropicalgin	1 (1–2) 1.31 ± 0.48	0.714	2 (2–3) 2.25 ± 0.45	0.151	2 (const.)	<0.001 * b	3 (const.)	0.035 * b
MB wax 1	1 (1–2) 1.13 ± 0.34	0.108	2 (const.)	0.001 * b	1 (const.)	0.035 * b	2.50 (2–3) 2.50 ± 0.52	0.151
Zetaplus	2 (2–3) 2.06 ± 0.25	<0.001 * b	3 (2–3) 2.88 ± 0.34	0.024 * b	2 (const.)	<0.001 * b	3 (const.)	0.035 * b
Vonflex S Putty	2 (2–4) 2.88 ± 0.50	<0.001 * b	4 (const.)	<0.001 * b	3 (const.)	<0.001 * b	4 (const.)	<0.001 * b
COE- PAK	1 (1–2) 1.19 ± 0.40	0.246	2 (const.)	0.001 * b	2 (1–2) 1.75 ± 0.45	0.005 * b	2 (const.)	<0.001 * b

OIQ = overall image quality; Median (min-max), Mean ± SD, constant = const; a = comparisons among all models, b = post hoc analysis comparing each of the models containing an overlay with the control, * statistically significant.

**Table 3:** Comparison of the models in terms of spherical marker visibility across CT protocols and reconstruction modalities.

	Low-Dose Protocol				Standard Protocol			
Reconstruction	Primary		O-MAR		Primary		O-MAR	
	SMV Score	*p*	SMV Score	*p*	SMV Score	*p*	SMV Score	*p*
Model		<0.001 * a		<0.001 * a		<0.001 * a		<0.001 * a
Control	2.50 (2–3) 2.50 ± 0.52		3 (3–4) 3.25 ± 0.45		2 (2–3) 2.25 ± 0.45		3 (3–4) 3.06 ± 0.25	
Elite HD+	2 (2–3) 2.38 ± 0.50	0.483	3 (const.)	0.035 * b	2 (const.)	0.035 * b	3 (const.)	0.317
Tropicalgin	2 (const.)	0.001 * b	2 (const.)	<0.001 * b	2 (const.)	0.035 * b	3 (const.)	0.317
MB wax 1	2 (2–3) 2.13 ± 0.34	0.024 * b	3 (const.)	0.035 * b	2 (const.)	0.035 * b	3 (const.)	0.317
Zetaplus	2 (2–3) 2.31 ± 0.48	0.288	3 (3–4) 3.25 ± 0.45	1.000	2 (const.)	0.035 * b	3 (const.)	0.317
Vonflex S Putty	3 (const.)	0.001 * b	4 (3–4) 3.63 ± 0.50	0.035 * b	2 (2–3) 2.19 ± 0.40	0.674	3 (3–4) 3.13 ± 0.34	0.551
COE- PAK	2 (const.)	0.001 * b	2 (2–3) 2.25 ± 0.45	<0.001 * b	2 (2–3) 2.06 ± 0.25	0.151	2 (2–3) 2.06 ± 0.25	<0.001 * b

SMV = spherical marker visibility; Median (min-max), Mean ± SD, constant = const.; a = comparisons among all the models, b = post hoc analysis comparing each of the models containing an overlay with the control, * statistically significant.

## Data Availability

The original contributions presented in this study are included in the article. Further inquiries can be directed to the corresponding authors.

## Abbreviations

The following abbreviations are used in this manuscript:

MDCT	Multidetector Computed Tomography
CBCT	Cone Beam Computed Tomography
MRI	Magnetic Resonance Imaging
O-MAR	Orthopedic Metal artifact reduction
AI	Artifact index
SD	Standard deviation
OIQ	Overall image quality
SMV	Spherical marker visibility
